# Two cases of sudden leaflet dehiscence after transcatheter aortic valve replacement

**DOI:** 10.1186/s44215-022-00011-4

**Published:** 2022-11-08

**Authors:** Yoshio Arai, Akira Marui, Atsushi Nagasawa, Nobuhisa Ohno

**Affiliations:** 1 Department of Cardiovascular Surgery, Tenri Hospital, 200 Mishima-cho, Tenri, Nara, 632-8552 Japan; 2 Department of Cardiovascular Surgery, Kokura Memorial Hospital, 3-2-1 Asano, Kokurakita-ku, Kitakyushu City, Fukuoka, 802-8555 Japan

**Keywords:** Transcatheter aortic valve replacement, Structural valve failure, Prosthetic valve deterioration, Reoperation

## Abstract

**Background:**

Transcatheter aortic valve replacement (TAVR) has caused a paradigm shift in the treatment of severe aortic stenosis. Although less invasive and good early results of TAVR have been reported, the long-term durability of the transcatheter aortic valve is still unclear.

**Case presentation:**

We performed an emergent surgical aortic valve replacement (SAVR) for two cases of acute heart failure due to sudden transcatheter aortic valve dehiscence after 7 or 6 years of primary TAVR. In both cases, transthoracic echocardiography revealed severe transvalvular regurgitation of the transcatheter aortic valve. Intraoperative findings revealed dehiscence on both sides of the anatomical non-coronary cusp without evident signs of degeneration, such as thickening, calcification, or infection. The postoperative course of the cases was uneventful, and the patients were discharged home on days 20 and 48 after the reoperation.

**Conclusions:**

Although the cause of the valvular disease is unknown, we are seriously concerned that the number of similar cases will increase in the future. We should be cautious in expanding the application of TAVR without evidence of long-term safety.

## Background

Transcatheter aortic valve replacement (TAVR) has caused a paradigm shift in the treatment of severe aortic stenosis (AS). Although less invasive and good early results of TAVR have been reported, the long-term durability of the transcatheter aortic valve is still unclear. We experienced two cases of surgical aortic valve replacement (SAVR) for acute heart failure due to sudden transcatheter aortic valve dehiscence mid-term after primary TAVR.

## Case presentation

### Case 1

**Fig. 1:**
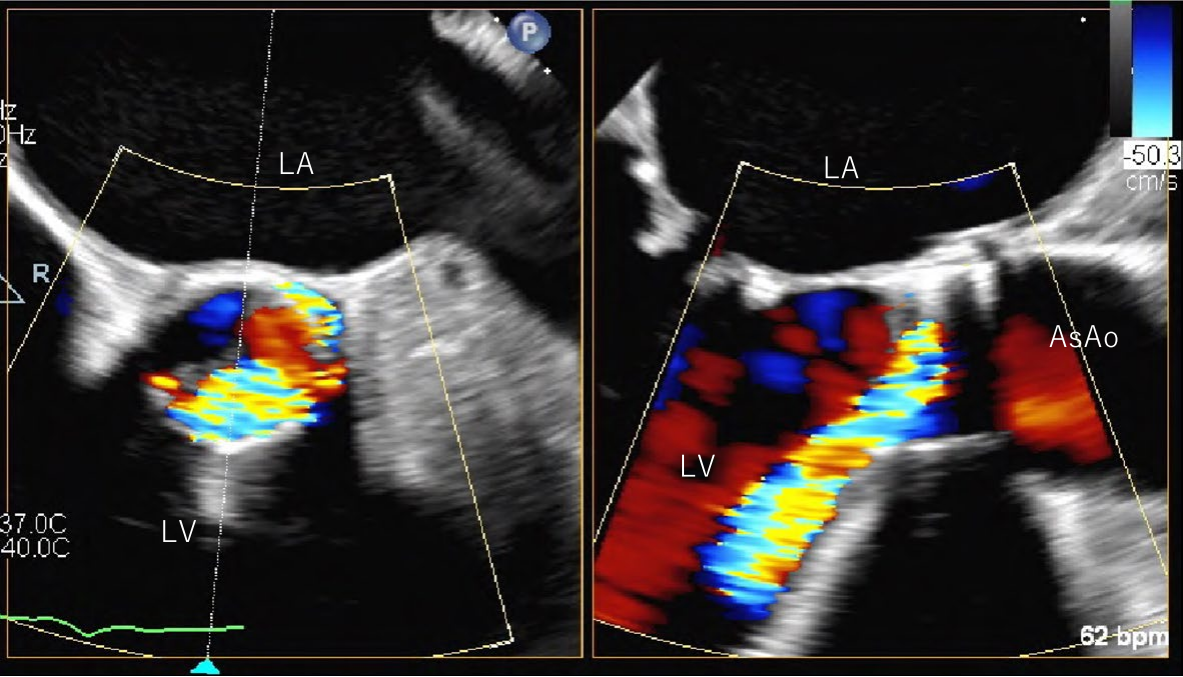
Preoperative transesophageal echocardiographic view of case 1

**Fig. 2:**
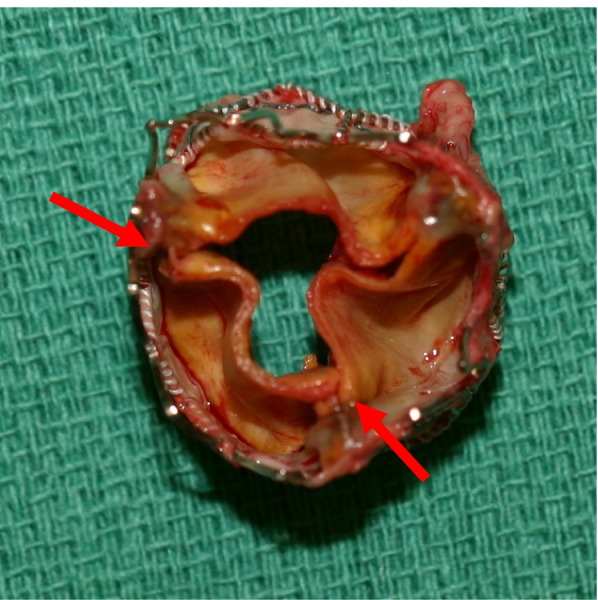
Photograph of the extraction transcatheter heart valve of case 1

A 77-year-old man with a history of rheumatic fever underwent transfemoral TAVR (SAPIEN XT 26 mm) accompanied by percutaneous transseptal mitral commissurotomy for AS and moderate mitral valve stenosis with severe calcified aorta. His postoperative course was uneventful. He underwent pacemaker implantation 1 year after a primary TAVR due to atrial fibrillation with bradycardia. Thereafter, regular follow-up with transthoracic echocardiography (TTE) revealed no signs of aortic valve dysfunction. Six years after the primary TAVR (83 years old), the patient was brought to the emergency department with sudden chest pain and severe dyspnea. Chest radiography revealed severe pulmonary edema. Transthoracic echocardiography showed previously unobserved severe aortic regurgitation (AR) at the anatomical non-coronary cusp (Fig. 1). Emergent surgical aortic valve replacement (SAVR) was planned because of the medically uncontrollable acute AR. Median sternotomy was performed, cardiopulmonary bypass was established, and cardiac arrest was achieved by retrograde cardioplegia. Operative findings revealed leaflet dehiscence of the prosthetic valve located at one of the cusps, deviating to the left ventricle (Fig. 2). There were less valvular organic changes, such as calcification and degeneration, and no obvious signs of infection. The valve was removed without damaging the perivalvular structures. Next, the right side of the left atrium was incised, and the left atrial appendage was closed. Mitral valve replacement (Epic 29 mm: St. Jude Medical, St. Paul, MN) was then performed, followed by SAVR using a bioprosthetic valve (Avalus 23 mm: Medtronic, Santa Rosa, CA), and finally, a tricuspid ring annuloplasty (Tri-Ad 30 mm: Medtronic, Santa Rosa, CA) was performed. Weaning from the cardiopulmonary bypass was smooth and easy. Postoperatively, the patient required continuous hemodialysis for 4 days. The patient was transferred to the general ward on the 13th postoperative day and discharged on postoperative day 48.

### Case 2

**Fig. 3:**
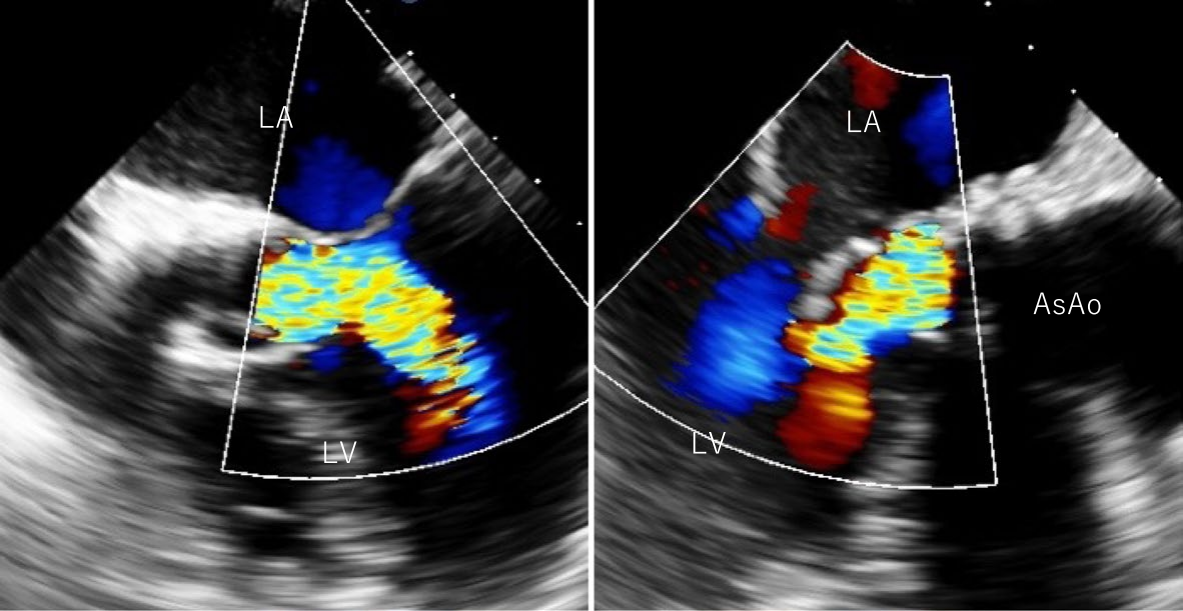
Intraoperative transesophageal echocardiographic view of case 2

**Fig. 4:**
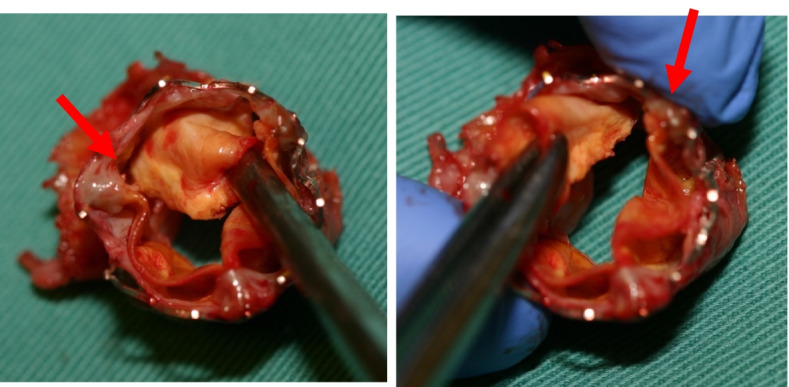
Photograph of the extraction transcatheter heart valve of case 2

A 72-year-old man with a history of coronary artery bypass surgery (60 and 62 years old) underwent transfemoral TAVR (SAPIEN XT 26 mm). His postoperative course was uneventful. Regular follow-up with TTE revealed no signs of aortic valve dysfunction. Six years after the primary TAVR (79 years old), the patient suddenly experienced chest tightness and respiratory distress. The patient was transported to a local hospital. He was diagnosed with acute heart failure without significant electrocardiographic changes or elevated cardiac enzyme levels. Transthoracic echocardiography showed moderate-to-severe AR in the prosthetic valve. Coronary angiography revealed 90% stenosis of the great saphenous vein bypass graft in the posterior descending branch of the right coronary artery, and a drug-eluting stent was implanted in the same area. However, the patient developed decompensated heart failure, which resulted in marked pulmonary congestion. Emergent surgical aortic valve replacement (SAVR) was planned because of the medically uncontrollable acute AR. The patient was transferred to our institution for an emergency surgery. On admission, TTE revealed a severe transvalvular AR (Fig. 3). Median sternotomy was performed, cardiopulmonary bypass was established, and cardiac arrest was achieved by retrograde cardioplegia. Operative findings revealed leaflet dehiscence of the prosthetic valve located at one of the cusps, deviating to the left ventricle (Fig. 4). There were less valvular organic changes, such as calcification, degeneration, and no obvious signs of infection. The valve was removed without damaging the perivalvular structures, and SAVR was performed using a bioprosthetic valve (Inspiris Resilia 21 mm: Carpentier-Edwards, Irvine, CA). Weaning from the cardiopulmonary bypass was smooth and easy. The patient’s postoperative course was uneventful. He was transferred to the referral hospital for rehabilitation on postoperative day 13.

## Discussion and conclusions

Although there have been some reports of self-expanding valvular rupture early after a primary TAVR [1], there has been, within the scope of our search, no report on emergent SAVR for acute AR due to sudden dehiscence of the prosthetic valve leaflet of balloon-expandable long-term after a primary TAVR. From October 2013, when insurance coverage of TAVR began in Japan, until April 2021, when the procedure was performed in this reported case, we performed TAVR for 1303 patients at our institution alone. During this period, only six (0.5%) patients had unscheduled reoperation after TAVR, requiring a repeat surgery. Prosthetic valve cusp rupture, as described above, has also been reported in bioprosthetic valves implanted via SAVR. One study reported a valvular leaflet rupture of the Carpentier-Edwards Magna Ease (Edwards Lifesciences Corp., Irvine, CA, USA) [2], which is similar to SAPIEN XT (Edwards Lifesciences), 8.7 years postoperatively. During the development of the SAPIEN XT, endurance tests were conducted for 200 million beats, equivalent to 25 years of heartbeats, and the results were reported to be comparable to those of Carpentier-Edwards Magna Ease [3]. The SAPIEN XT transcatheter heart valves (THV) removed in the current surgeries were validated by the Edwards Lifesciences Corporation, and manufacturing problems have not been reported. In both cases, the THV was implanted oversized and post-BAV was performed, which may have placed excessive stress on the THV. Unfortunately, no histological evaluation was performed. It is generally stated that bioprosthetic valves may deteriorate over time due to the deposition of calcareous components, growth of autologous tissue, and adhesion of thrombi [4].

The difference in the sewing method between the SAVR and TAVR valve leaflets may possibly have an effect, but unfortunately, a public documentation about how to fix SAVR and TAVR prosthesis leaflets is not provided by the manufacturing company.

Additionally, it has been reported that the durability of surgical bioprosthetic valves is inadequate for younger patients compared to older patients [5], and the current recommendation of preferring older patients for TAVR is appropriate until the long-term results become clearer.

TAVR valve removal requires care, and we carefully detached the frame and intima from the top of the transcatheter aortic valve in a circumferential fashion, bending the valve as we detached it. Nakazato et al. reported an interesting method of removing the valve by cutting the frame longitudinally with nippers to release the radial force [6].

The cause of acute AR in these cases is unclear. Both patients underwent regular follow-ups with TTE and periodic medication adjustments. Patient 1 had undergone a scheduled follow-up 10 days prior to symptom onset. At that time, the patient had no symptoms of heart failure, TTE showed mild paravalvular AR related to SAPIEN XT THV, and the examining physician considered the patient to be doing well. The patient presented with acute heart failure and was suspected to have acute-onset severe prosthetic valve dysfunction. We surmised that the sudden rupture of the prosthetic valve leaflet and the resulting gap caused severe AR, leading to acute heart failure. Patient 2 had no heart failure symptoms 1 year prior to onset, and on TTE, there was only mild paravalvular AR related to SAPIEN XT. Six months prior to the TTE, the paravalvular AR was rated as mild to moderate. During the next 6 months, the valve leaflet ruptured, which caused severe AR, leading to acute heart failure. Additionally, the worsening of AR from mild to moderate at 1-year and 6-month follow-up may have been a predictor of this event. Placement of a THV inside a transcatheter aortic valve (TAV-in-TAV) has been reported as an effective procedure [7]. Since this was a high-risk patient with a history of twice coronary artery bypass grafting procedures, we would have preferred performing TAV-in-TAV. We had to select SAVR because TAV-in-TAV is not covered by insurance in Japan at present. Approval for TAV-in-TAV in Japan is anticipated.

On the other hand, a new problem has occurred. TAV-in-TAV increased the risks for technically impossible coronary access or coronary obstruction, valve thrombosis, and aortic regurgitation due to perivalvular leakage. Mauler-Wittwer et al. reported that TAV-in-TAV was not suitable in 30% of cases due to anatomical limitations and that it was not always feasible [8]. In a report on aortic valve reintervention after TAVR in a real-world multicenter registry, Fukuhara et al. reported that the number of reinterventions increased and TAVR explants among all reintervention procedures are increasing year by year [9].

Currently, SAPIEN XT is not used in Japan, and a modified version, SAPIEN 3, is in use. Pibarot et al. reported that SAPIEN XT had more events than surgical valves over 5 years, but SAPIEN 3 was comparable to surgical valves [10]. SAPIEN 3 is expected to have good durability. Our institution has never experienced leaflet dehiscence with SAPIEN 3. However, it has been only 5 years since the introduction of SAPIEN 3 in Japan, and no conclusion can be drawn. Continued careful follow-up is required.

In conclusion, although the cause of the valvular disease is unknown, we are seriously concerned that the number of similar cases will increase in the future. We should be cautious in expanding the indications of TAVI without evidence of long-term safety.

## Data Availability

Data sharing is not applicable to this article, as no datasets were generated or analyzed during the current study.

## Abbreviations

TAVR: Transcatheter aortic valve replacement
TTE: Transthoracic echocardiography
AR: Aortic regurgitation
SAVR: Surgical aortic valve replacement
THV: Transcatheter heart valve
TAV: Transcatheter aortic valve
